# Air-liquid interface culture for live analysis of zebrafish vertebral growth

**DOI:** 10.17912/micropub.biology.001199

**Published:** 2025-03-04

**Authors:** Shin Sato, Misaki Sakashita, Ryo Kagami, Naoyuki Wada

**Affiliations:** 1 Department of Applied Biological Science, Tokyo University of Science, Noda, Chiba, Japan

## Abstract

Fish vertebrae change their shapes from simple cylinders to complex amphicoelous hourglass-like forms with lateral ridges as they grow. This growth process is difficult to observe *in vivo* due to the thickening of the muscles surrounding the vertebrae. In this study, we applied air-liquid interface culture to isolated zebrafish vertebrae to observe vertebral growth *in vitro* . The mineralization patterns of the cultured vertebrae were similar to those of *in vivo* growth, reproducing vertebral growth. This study is the first report to ensure live analysis of fish vertebral growth from late larval stages and the related cell behavior.

**Figure 1. Application of air-liquid interface culture to zebrafish vertebrae to observe the growth of vertebral centra, arches, and spines:**
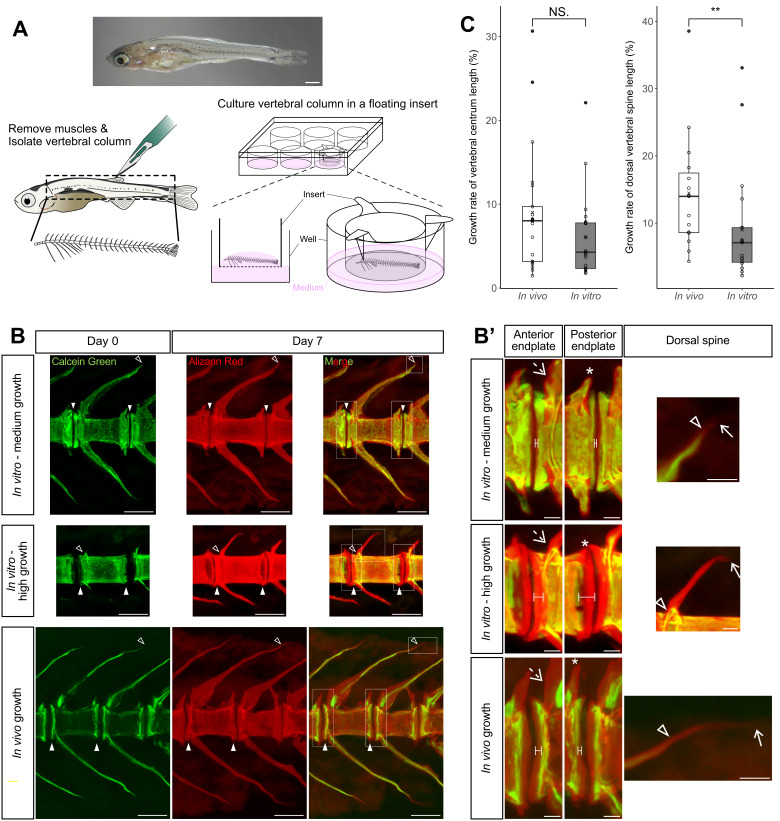
(A) The culture method of zebrafish vertebrae. We used late larval zebrafish (upper panel, SL 8.5 mm). First, we isolated the vertebral column from an individual by removing the surrounding muscles (lower left). We then placed the vertebral column on the porous insert membrane immersed in the medium and incubated the well plate at 28 °C for 7 days (lower right). (B) Double labeling of fluorescent dyes to observe the change of mineralized area during *in vitro* and *in vivo* growth for 7 days. The vertebrae in the images were the 5th from the last, excluding the urostyle. White and line arrowheads indicate the edges of the vertebral centra and dorsal vertebral spines stained with calcein green, respectively. The area stained with alizarin red extends beyond the area stained with calcein green, as indicated by the arrowheads, suggesting bone growth during the experiment. The fluorescence intensity of *in vivo* calcein green staining was weak on the lateral sides of the vertebral centrum and strong on the vertebral centrum endplates and parts of the vertebral arches and spines, whereas that of *in vitro* calcein green staining was almost uniform throughout the vertebral body and spines. (B’) Magnified images of the anterior and posterior vertebral centrum endplates (left and middle columns) and dorsal vertebral spines (right column) of the 5th vertebrae from the last marked with the rectangles in (B). Dimension lines indicate the differences between calcein green and alizarin red staining at the edges of the vertebral centra. Dashed arrows and asterisks indicate the dorsal pre-zygapophyses and post-zygapophyses, respectively. Solid arrows indicate the edges of the dorsal vertebral spines stained with alizarin red. The vertebrae whose dorsal spines were not stained with calcein green were excluded from the measurements (middle right panel). (C) Comparison of *in vivo* and *in vitro* growth rates. The sample size was 21 *in vivo* and 21 *in vitro* for vertebral centra lengths and 15 *in vivo* and 19 *in vitro* for dorsal vertebral spines. The circles in the box plots represent individual data points, and the crosses represent the means. The mean growth rate of vertebral centrum length was 9.02% (SD 7.23%) *in vivo* and 6.09% (SD 4.82%) *in vitro* . The mean growth rate of vertebral dorsal spine length was 14.46% (SD 8.42%) *in vivo* and 9.00% (SD 8.15%) *in vitro* . The high-value outliers (black circles) were defined as high growth. ** *P* < 0.01, Mann-Whitney *U* test. Scale bars: (A) 1 mm, (B) 100 µm, (B’) 20 µm. SD, standard deviation.

## Description

Vertebral formation is an essential developmental process for supporting the body axis in vertebrates. In teleosts, vertebral structure dynamically changes during growth from a thin cylindrical bone called the chordacentrum in early larval stages to an hourglass-shaped autocentrum with elongated vertebral arches and spines (Grotmol et al., 2003; Nordvik et al., 2005) and species-specific complex lateral structures (Laerm, 1976; Sakashita et al., 2019) in adult stages. Previous observations in early larval fish have shown that osteoblasts are distributed at the vertebral arches and the anterior and posterior edges of the chordacentrum (Azetsu et al., 2017; Inohaya et al., 2007; Spoorendonk et al., 2008) , suggesting that osteoblasts deposit bone at the edges to elongate the vertebral arches, spines, and centra. However, the subsequent growth process to form the adult vertebrae has rarely been explained because *in vivo* imaging of larger vertebrae is difficult. The vertebrae of early larval zebrafish and medaka are easy to observe because their bodies are transparent, but the muscles and skin surrounding the vertebrae become thick from late larval to adult stages. For this reason, the later development of teleost vertebrae has mainly been described by observing histological sections at different developmental stages (Nordvik et al., 2005; Sakashita et al., 2024) . However, the descriptions based on these histological sections have a gap in understanding the detailed spatiotemporal changes in the vertebrae. Therefore, a method to visualize vertebrae during growth is needed to explain vertebral structural changes and related cell activities in late developmental stages.

To date, organ culture has been developed to observe the dynamic process of bone formation in fish at the late stages of development, mainly using scales, fins, and craniums (LeClair, 2021) . However, the application of organ culture to fish vertebrae has not been reported. In contrast, in mammals, mouse vertebral centra are elongated using the air-liquid interface culture (Aono & Hirai, 2011) . The air-liquid interface culture is one of the popular methods for organ culture, in which bones on the insert membrane are exposed to medium and air to provide sufficient oxygen easily. Based on these, we hypothesized that air-liquid interface culture could be applied to observe the dynamic changes in vertebral growth of fish. Therefore, we tried to combine the conventional organ culture methods for fish bones and the air-liquid interface method to culture fish vertebrae. For stable organ culture experiments, it is essential to verify how the culture system functions and mimics *in vivo* growth. In this study, we constructed the air-liquid interface culture system of fish vertebrae and evaluated the efficiency of the culture system.

We cultured late larval zebrafish vertebrae on a porous filter insert in 6-well plates ( Figure 1A ) for 7 days and compared vertebral growth with *in vivo* growth for the same period. We set the culture period at 7 days to collect a sufficient number of samples for statistical analysis, although the culture period in this system can be extended to observe further vertebral growth because no contamination was observed after 7 days of culture. To evaluate the efficiency of the culture, we visualized the progression of mineralization during growth by double fluorescence labeling of the bones with Calcein Green and Alizarin Red S. In the double labeling, the vertebrae were stained green before the culture and red at the end of the culture. The mineralized area before the culture is stained both green and red, and the mineralized area during the culture is observed as red only. Comparison between *in vitro* and *in vivo* growth using this fluorescent labeling showed that the position and direction of mineralization were similar ( Figure 1B ). The anterior and posterior edges of the vertebral centra were elongated anteriorly and posteriorly by bone mineralization (Figures 1B and 1B’, n=21/21 *in vitro* , n=22/22 *in vivo* ). Bone elongation was also observed in the pre-zygapophyses ( Figure 1B ’, n=21/21 *in vitro* , n=21/22 *in vivo* ) and post-zygapophyses ( Figure 1B ’, n=20/21 *in vitro* , n=14/22 *in vivo* ). The tips of the vertebral spines were obliquely elongated (Figures 1B and 1B’, n=19/21 *in vitro* , n=15/22 *in vivo* ). These results indicate that zebrafish vertebrae can grow under air-liquid interface culture.

We further compared bone growth rates *in vitro* and *in vivo* by measuring the lengths of the vertebral centra and dorsal spines ( Figure 1C ). Because both growth rate data deviated from a normal distribution and had multiple high-value outliers, we performed a Mann-Whitney *U* test and compared the median growth rates between *in vitro* and *in vivo* growth. For vertebral centra lengths, no statistically significant difference was found between *in vitro* and *in vivo* growth rates. However, the median growth rate *in vitro* was lower than *in vivo* . The median *in vitro* growth rate was 4.28%, which was 53.2% of the median *in vivo* growth rate of 8.02%. For vertebral dorsal spines, *in vitro* growth rates were statistically significantly lower than *in vivo* . The median *in vitro* growth rate was 7.09%, which was 50.7% of the median *in vivo* growth rate of 14.0%. These results suggest that this culture system can reproduce at least half of the *in vivo* growth rates for 7 days.

The growth rates of the vertebral centra and dorsal vertebral spines did not correlate with the standard length of the fish. In both *in vivo* and *in vitro* groups, the coefficient of determination R ^2^ in the linear approximation was less than 0.1. In several vertebrae, the elongations of the vertebral centra lengths were more pronounced than in other vertebrae (high growth in Figures 1B and 1B’), and the growth rates were 30.7 and 24.6% *in vivo* and 22.1% *in vitro* ( Figure 1C left). Also, significant elongations of the dorsal vertebral spines were observed, and the growth rates were 38.5% *in vivo* and 33.1 and 27.6% *in vitro* ( Figure 1C right). These pronounced elongations of the vertebral centra and dorsal spines were observed *in vivo* and *in vitro* , suggesting that this culture system can also reproduce the variation in zebrafish vertebral growth. Because the culture medium consisted only of essential medium with serum and growth-promoting reagents were minimal, the degree of vertebral growth in culture probably depended on the condition of the vertebrae before culture. In the vertebrae that elongated significantly, the intervertebral regions between the vertebral centra were wide before *in vitro* culture and *in vivo* growth. These wide intervertebral regions may be rich in osteoid, which may strongly promote mineralization at the edges of the vertebral centra during growth.

To date, no method has been established for live analysis of later vertebral growth in teleosts. For this reason, the dynamic vertebral growth in later developmental stages remains unclear. This study is the first report to ensure the live analysis of zebrafish vertebral growth by reproducing the *in vivo* growth on the air-liquid interface culture. Since the vertebral growth in culture indicates the presence of cells actively forming bone tissue, we can analyze the cellular activities and responses to physiological signals for vertebral growth by using transgenic zebrafish in which the cells are visualized with fluorescence, trying any reagent treatment, performing experimental manipulation, etc. In recent studies, zebrafish have been increasingly used as a convenient model organism to understand human spinal diseases such as scoliosis and lordosis (Dietrich et al., 2021; Hayes et al., 2014; Printzi et al., 2021; Ramli et al., 2023). The culture system will help us to deeply understand not only the development of fish vertebrae, but also the mechanism of spinal diseases.

## Methods

**Zebrafish**

All experiments in this study were performed under the guidelines and approved protocols for animal care and use at Tokyo University of Science. AB strains were bred as wild-type lines following Nüsslein-Volhard and Dahm (2002). Standard length (SL) was measured according to Parichy et al. (2009). We used 21 and 22 late larvae with SL of 6.2–10.5 mm for *in vitro* and *in vivo* experiments, respectively. To reduce contaminants in the fish gut, both the fish for *in vitro* culture and the fish reared *in vivo* for the control were isolated from communal housing and fasted for 24 hours prior to the experiment.

**Vertebrae culture with double fluorescent bone labeling**

We constructed the culture system following Miyake and Hall (1994). Zebrafish were euthanized with ~160 mg/L tricaine solution (Wako). The head and internal organs were removed with tweezers and knives, and the body surface was wiped with a paper towel moistened with 70% ethanol. Then, the skin and muscles were removed with tweezers and knives in phosphate-buffered saline (PBS) containing 1% Antibiotic-Antimycotic (100X, Gibco) to isolate the vertebral column. Tweezers and knives were sterilized with 70% ethanol and heat sterilized after each use during dissection. We prepared the culture medium by mixing 10% fetal bovine serum (FBS, Biological Industries) in Leibovitz’s L-15 medium (Wako) and adding 1% Antibiotic-Antimycotic (100X). We added 2 mL of culture medium to each well of 6-well plates (Greiner Bio-One). After washing the vertebral column twice in PBS containing 1% Antibiotic-Antimycotic (100X), we immersed the vertebral column in 0.01% (w/v) Calcein Green (DOJINDO, product code: C001, CAS No. 1461-15-0) solution for 20 minutes. Then, the vertebral column was washed in PBS containing 1% Antibiotic-Antimycotic and placed on ThinCert® cell culture inserts (Greiner Bio-One, Item No. 657610, pore diameter: 1 µm) in the culture medium of the well plates for the air-liquid interface method. The plates were covered with aluminum foil to prevent fluorescence fading. We incubated the plates at 28 °C in a hybridization oven for 7 days. We replaced 1 mL of medium with fresh medium every other day. At the time of medium replacement, we observed green fluorescence on the vertebrae using a culture microscope CKX53 (OLYMPUS) to confirm that calcein green staining was maintained.

After culturing, the vertebrae were delipidated in 1mL 10% (v/v) 1,2-hexanediol by slow shaking at room temperature (23–26 °C) for 20 minutes for tissue clearing, based on Inoue et al. (2019). The 1,2-hexanediol was diluted in PBS. After a brief wash in MilliQ for no longer than 1 minute, the vertebrae were stained in 0.01% (w/v) Alizarin Red S (Wako, Product number: 011-01192, CAS No. 130-22-3) solution for 20 minutes and then washed briefly in MilliQ. The calcein green and alizarin red were dissolved in MilliQ. Finally, the vertebrae were cleared by slow shaking at room temperature (23–26 °C) for more than 16 hours in RapiClear 1.52 (SunJin Lab).

Double-stained vertebrae were photographed using z-stack imaging on an all-in-one fluorescence microscope BZ-X810 (KEYENCE). The sequential z-stack images were combined into a single image using the full focus function and used for measurements.

***In vivo* growth with double fluorescence labeling**

To stain the vertebrae of live zebrafish, we allowed the individuals to swim in 0.01% calcein green solution for 20 minutes. After 7 days of rearing in tank water, the calcein green-stained fish were euthanized, and the vertebral column was isolated. Isolated vertebrae were delipidated with 1,2-hexanediol, stained with alizarin red, and cleared in RapiClear, as were cultured vertebrae.

**Evaluation of bone growth rates**

The lengths of the vertebral centra and dorsal vertebral spines were measured using the Straight line selection tool and the Segmented line selection tool of ImageJ ( https://imagej.net/ij/ ), respectively. We drew the line at the midline on the lateral side of the alizarin red-stained vertebral centrum and measured its length, then moved the end along the line to the edge of the calcein green-stained area and measured it. We also drew the segmented line along the alizarin red-stained dorsal vertebral spine and measured its length, then adjusted the segment to measure the length of the calcein green-stained area. Growth rates were calculated by dividing the difference in length between the areas stained with alizarin red and calcein green by the length of the area stained with calcein green.

For each individual, growth rates were calculated on the 4th to 6th vertebrae from the last, excluding the urostyle. The average of the three vertebrae was used for statistical analysis. Seven fish from the *in vivo* experiments, two from the *in vitro* experiments whose vertebral spines were not stained with calcein green, and one from the *in vivo* experiment whose vertebral centra were distorted were excluded from the measurement. For statistical analysis, we used the wilcox.test function of R for the Mann-Whitney *U* test.
